# The GCaMP3 – A GFP-based calcium sensor for imaging calcium dynamics in the human malaria parasite *Plasmodium falciparum*

**DOI:** 10.1016/j.mex.2014.08.005

**Published:** 2014-08-27

**Authors:** Lucas Borges-Pereira, Bruna R.K.L. Campos, Celia R.S. Garcia

**Affiliations:** a Departamento de Fisiologia, Instituto de Biociências, Universidade de São Paulo, São Paulo, Brazil; b Departamento de Parasitologia, Instituto de Ciências Biomédicas, Universidade de São Paulo, Brazil

**Keywords:** Malaria, GECIs, GFP, Calcium, Drug screening, *Plasmodium falciparum*, GCaMP3

## Abstract

Calcium (Ca^2+^) signaling pathways are vital for all eukaryotic cells. It is well established that changes in Ca^2+^ concentration can modulate several physiological processes such as muscle contraction, neurotransmitter secretion and metabolic regulation (Giacomello et al. (2007) [1], Rizzuto and Pozzan (2003) [2]). In the complex life cycle of *Plasmodium falciparum*, the causative agent of human malaria, Ca^2+^ is involved in the processes of protein secretion, motility, cell invasion, cell progression and parasite egress from red blood cells (RBCs) (Koyama et al. (2009) [3]).

The generation of *P. falciparum* expressing genetically encoded calcium indicators (GECIs) represents an innovation in the study of calcium signaling. This development will provide new insight on calcium homeostasis and signaling in *P. falciparum*. In addition, these novel transgenic parasites, PfGCaMP3, is a useful tool for screening and identifying new classes of compounds with anti-malarial activity. This represents a possibility of interfering with signaling pathways controlling parasite growth and development. Our new method differs from previous loading protocols (Garcia et al. (1996) [4]; Beraldo et al. (2007) [5]) since:

• It provides a novel method for imaging calcium fluctuations in the cytosol of *P. falciparum*, without signal interference from the host cell and invasive loading protocols.
• This technique could also be expanded for imaging calcium in different subcellular compartments.
• It will be helpful in the development of novel antimalarials capable of disrupting calcium homeostasis during the intraerythrocytic cycle of *P. falciparum*.

## Graphical abstract

**Figure:**
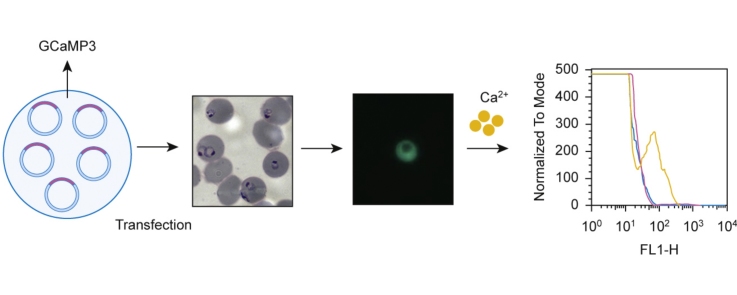

## Method details

Calcium (Ca^2+^) is a ubiquitous signaling molecule and acts in several physiological processes from mammalian cells to parasites (Rizzuto and Pozzan 2003 [2]; Giacomello, Drago et al. 2007 [1]; Koyama, Chakrabarti et al. 2009 [3]). To monitor the calcium fluctuations in *Plasmodium falciparum* invasive loading protocols are used and do not allow discrimination of signals from the host cell and intracellular parasites (Garcia, Dluzewski et al. 1996 [4]; Beraldo, Mikoshiba et al. 2007 [5]). Generation of transgenic *Plasmodium falciparum* expressing GECI represents an innovation, allowing monitoring calcium fluctuations in the cytosol of *P. falciparum* without invasive loading protocols and interference from the host cell.

Construction of transgenic *Plasmodium falciparum* requires the following steps: (i) cloning of GCaMP3 gene into *P. falciparum* expression vector pDC, (ii) transfection of *P. falciparum* with the pDC/GCaMP3 constructs, (iii) selection of the transfected population, (iv) analyses of PfGCaMP3 calcium responses.

*Step 1*: Cloning of GCaMP3 gene into *P. falciparum* expression vector pDC.

The ORF encoding the GCaMP3 gene was amplified from the original mammalian expression plasmid (Addgene, kindly supplied by Dr. Silvia Moreno) using the specific primers: 5′ GGATCCATGGGTTCTCATCATCATCATC 3′ and 5′ GGATCCTTACTTCGCTGTCATCATTTGTAC 3′. The BamH I cleavage site was added in the 5′ end of both primers. Approximately 100 ng of plasmid was used in a PCR reaction in the following conditions: 94 °C/5′, 34 cycles of 95 °C/55″; 50 °C/60″ and 68 °C/180″ and a final step of 74 °C/5′. The amplicons, approximately 1.3 kb, were purified from agarose gel using PureLinkTM Quick Gel Extraction Kit (Invitrogen) according with the manufacture's protocols. The purified amplicons were then cloned into bacterial propagation vector pJET (Fermentas) and used to transform One Shot^®^ TOP10 Chemically Competent *E. coli* (Invitrogen). The transformant selection was made in LB agar plates at 37 °C for 20 h. Single colonies were grown in 5 mL of LB/ampicillin at 37 °C for 20 h at 180 rpm. Plasmid DNA was extracted with Wizard^®^ Plus SV Minipreps DNA Purification Systems (Promega).

To test for the presence of GCaMP3 gene, plasmid DNA was submitted to restriction analyses with the BamH I enzyme. The reaction was performed with approximately 10 U of enzyme at 37 °C for 2 h. All tested colonies had the GCaMP3 gene. The cloning confirmation was also obtained by sequencing reaction. The tested colonies presented a gene that was identical with the synthetic construct GCaMP3 gene (GI: 299818412) present in Basic Local Alignment Search Tool (BLAST^®^) website.

The GCaMP3 gene was then transferred to *P. falciparum* transfection plasmid pDC [6] (kindly supplied by Dr. Gerhard Wunderlich). For this purpose, pJET/GCaMP3 construct and pDC vector were submitted to a new restriction reaction with BamH I enzyme and the fragments, approximately 1.3 and 6 kb, corresponding to GCaMP3 and pDC respectively, were purified from the agarose gel as described above. The ligation reaction was carried out at 16 °C overnight and was used to transform chemically competent *E. coli* using the heat shock method. From the 12 colonies obtained, colonies 8 and 11 were positive for GCaMP3. The correct insertion of the gene was confirmed by restriction analyses with Xho I, since digestion with this enzyme results in fragments with different sizes depending on the orientation of the insert. Colonies 8 and 11 showed the GCaMP3 inserted in the correct orientation. We selected colony 11 for sequencing and the gene was identical with the synthetic construct GCaMP3 gene present in Basic Local Alignment Search Tool (BLAST^®^) website.

*Step 2*: Transfection of *P. falciparum* with the pDC/GCaMP3 constructs.

**Fig. 1:**
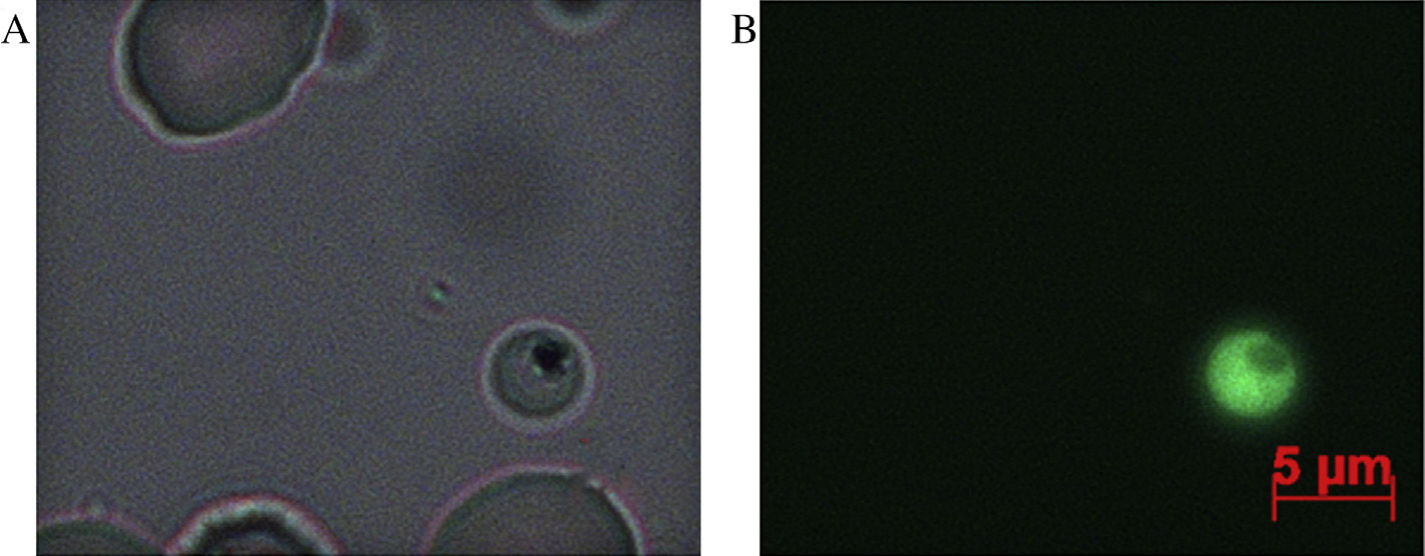
*P. falciparum*-infected RBC transfected with the calcium indicator GCaMP3. (A) Bright field and (B) fluorescence image of a parasite expressing GCaMP3. The images were acquired in an Axio Scope A.1 microscope (Zeiss) and analyzed with AxioVison 4.8 software. The objective used was a 100× N-Achroplan with oil immersion. Scale bar = 5 μm.

A synchronized ring culture of *P. falciparum* with a parasitemia of approximately 10% was submitted to electroporation with 50 μg of pDC/GCaMP3 constructs. Briefly, 200 μL of cells were resuspended in 500 μL of Cytomix (120 mM KCl, 0.15 mM CaCl_2_, 2 mM EGTA, 5 mM MgCl_2_, 10 mM K_2_HPO_4_/KH_2_PO_4_, 25 mM HEPES), the plasmid was added and the mixture was transferred into a 0.4-cm cuvette. The electroporation conditions were as follows: 2.5 kV, 25 μF and 200 Ω. The cuvette was placed on ice for 5 min. The cells were then transferred to a culture flask with 10 mL of culture medium (RPMI 1640 Gibco supplemented with 0.5% albumax, 2 g/L sodium bicarbonate, 40 mg/L gentamicin and 50 mg/L hypoxanthine) and placed in an incubator (5% CO_2_, 5% O_2_, and 90% N_2_). After 48 h 5 nM of WR99210 was added to the culture medium to select for transformants. The transfected parasites (PfGCaMP3) started to appear approximately after 2 weeks and the fluorescent parasites could be observed in a fluorescence microscope (Fig. 1).

*Step 3*: Selection and enrichment of the transfected population.

The transfected population was submitted to a cell sorting by flow cytometry to select those parasites with increased fluorescence intensity at 488 nm. The experiment was carried out in a FACSAria II™ cell sorter (BD Biosciences). The selected parasites were maintained for 1 week and then cloned by limiting dilution in 96-well plates (0.5 parasite/well). The parasites could be detected approximately 2 weeks after the limiting dilution by measuring the activity of LDH enzyme. LDH activity was measured with Malstat reagent (400 μL of Triton X-100 in 80 mL of deionized water, 4.00 g of l-lactate, 1.32 g of Tris buffer, 0.022 g of 3-acetylpyridine adenine dinucleotide (APAD), pH 9 in the final volume of 200 mL) and NBT/PES solution (0.160 g of nitro blue tetrazolium salt and 0.008 g phenazine ethosulfate in 100 mL of deionized water). Fifteen μL of the culture was taken from each well and added to a plate containing 100 μL of Malstat reagent and 25 μL of NBT/PES solution and the color development of the LDH plate was monitored colorimetrically at 620 nm [7]. From the total clones obtained, we selected one with an increased percentage of fluorescent parasites for further studies.

*Step 4*: Analyses of PfGCaMP3 calcium response.

**Fig. 2:**
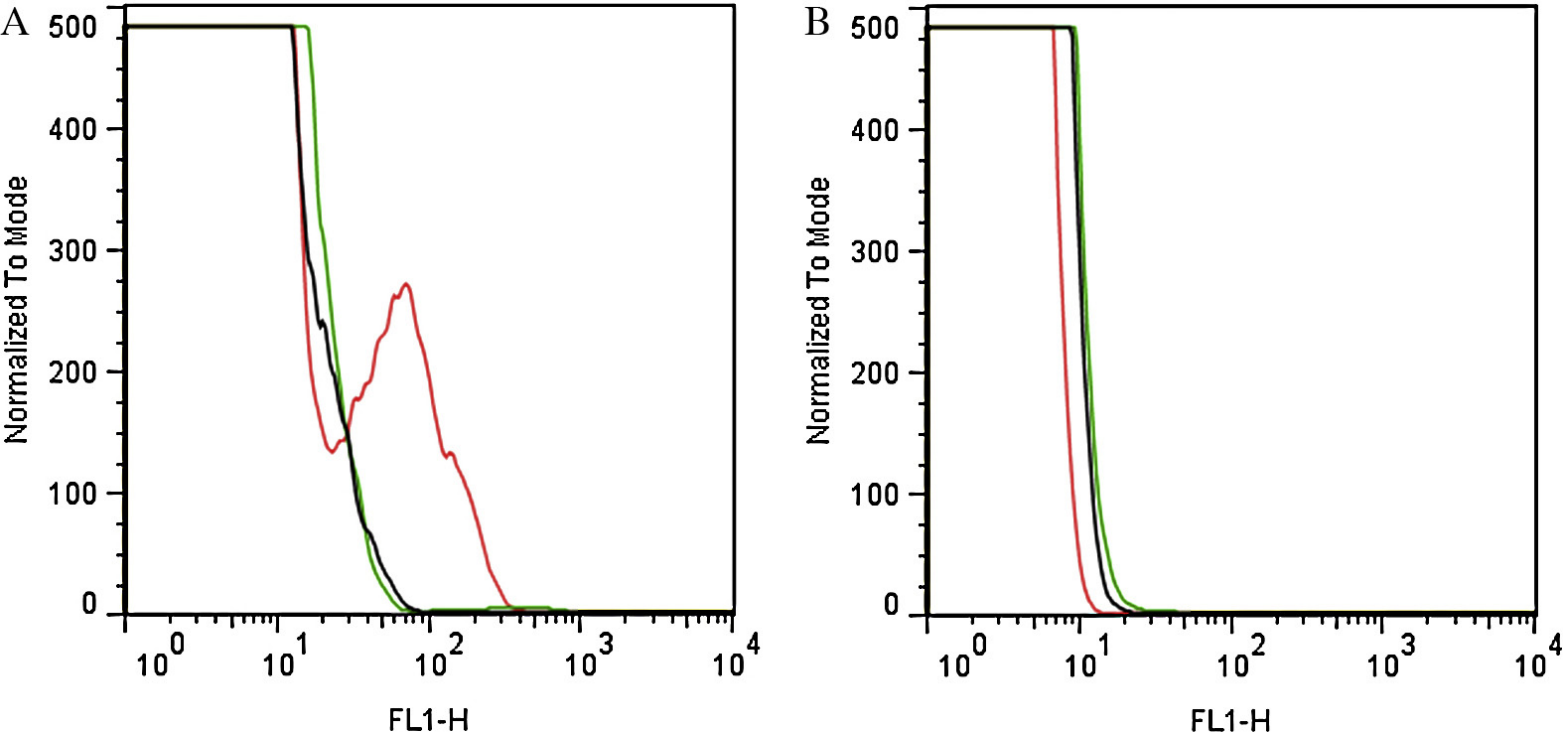
Calcium response of PfGCaMP3 parasites. (A) PfGCaMP3 and (B) wild type 3D7 parasites were stimulated with ionomycin (red) for 1 min. The increase in the fluorescence of the parasites was determined by flow cytometry analysis. FL1-H was used to detect GCaMP3 fluorescence emission at a wavelength of 530 nm. The overlaid histograms in green and black represent control without treatment and treated with DMSO, respectively.

To test the calcium response in the presence of the calcium ionophore ionomycin, we selected one clone from the 96 well plate, since this clone had a higher percentage of fluorescence parasites. Infected erythrocytes at trophozoite stage were centrifuged (2000 rpm, 5 min), and the pellet was washed twice and resuspended in 1 mL of buffer A (in mM: 116 NaCl, 5.4 KCl, 0.8 MgSO_4_, 5.5 d-glucose, 50 MOPS, 2 CaCl_2_). Two μM ionomycin was added and the cells were incubated for 1 min (Fig. 2). The calcium response was determined from dot plots [side scatter (SSC) versus fluorescence] of 10^5^ cells acquired on a FACS Calibur flow cytometer using CELLQUEST software (Becton & Dickinson). GCaMP3 was excited with a 488 nm argon laser and the fluorescence emission was collected at 520–530 nm [8].
